# Supplementary material for: The impact of human behavioral adaptation stratified by immune status on COVID-19 spread with application to South Korea
Source: PLoS One. 2026 Apr 6;21(4):e0345642. doi: 10.1371/journal.pone.0345642 (PMC13052854; doi:10.1371/journal.pone.0345642)
Supplement: Attachment — Submitted filename: Review_of____PONE_D_24_53603R1.pdf [file pone.0345642.s002.pdf]

# Review of "The impact of human behavioral adaptation stratified by immune status on COVID-19 spread with application to South Korea"

This manuscript presents a compartmental model incorporating immunity-based behavioral responses to COVID-19, calibrated to South Korean data (Feb-May 2022). The authors distinguish between "partially immune" and "susceptible" populations, modeling how their differing risk perceptions (prevalence vs. severity) affect vaccination/testing behaviors and transmission dynamics. Key findings suggest that increasing information reactivity in partially immune individuals could reduce peak cases by 33%, while shifting their risk perception toward severity could increase peaks by 57%.

1. While the authors justify retaining births/deaths and vaccination components (Response to Reviewer 1), the model remains highly parameterized. Consider explicitly demonstrating how removing less influential parameters (e.g., natural demographics) affects core outcomes to strengthen the justification.

2. The revisions to $g_1$ (prevalence) and $g_2$ (severity) improve balance (Lines 306-316), but the assumption that "severity-reactivity is twofold of prevalence-reactivity" lacks empirical grounding. Sensitivity analysis across a wider range of this ratio would strengthen robustness claims.

3. The addition of IU1/IU2 compartments (Response to Reviewer 1a) addresses recovery rate interpretation issues. However:

   - The assumption that A1/I1 individuals "do not contribute to transmission" (Lines 114-120) during pre-testing phases may underestimate asymptomatic spread, particularly for Omicron.
   - Figure 1 should explicitly label flows from IT1/IT2 to H to resolve the noted ambiguity (Response to Reviewer 1, Fig 1 comment).

4. The focus on "active cases" (IU1+IU2) as the primary outcome (Response to Reviewer 1, Fig 5/6 comment) is reasonable for transmission dynamics but limits public health applicability. Including supplementary analyses of hospitalization/death peaks would enhance policy relevance.

5. The recalibration after model revisions (e.g., $T_{\max} = 0.8$) is noted (Lines 300-305), but details on parameter estimation methods (e.g., objective functions, confidence intervals) remain insufficient. Consider adding a calibration flowchart or sensitivity matrices.

- Define "partially immune" operationally (e.g., antibody thresholds, vaccination status) in Methods.
- Adding $V$ and $N$ as arguments to $F_1, T_1, F_2, T_2$ (Response to Reviewer 1) improves clarity. Ensure consistency in later equations.
- The added note about vaccination-immunity delay (Lines 642-646) is valuable. Also acknowledge potential seasonality effects omitted from the 4-month calibration window.
- The statement (Lines 619-629) is adequate, but specify preprocessing steps for the Our World in Data dataset.

## Conclusion

The manuscript addresses a critical gap in modeling immunity-stratified behavioral responses. The revisions significantly improve model rigor, particularly regarding compartmental flows and message functions. With enhanced calibration transparency and expanded outcome metrics, this work could provide valuable insights for post-pandemic endemic management. I recommend acceptance after minor revisions.
