# Supplementary material for: The impact of human behavioral adaptation stratified by immune status on COVID-19 spread with application to South Korea
Source: PLoS One. 2026 Apr 6;21(4):e0345642. doi: 10.1371/journal.pone.0345642 (PMC13052854; doi:10.1371/journal.pone.0345642)
Supplement: Attachment — Submitted filename: Response to Reviewers.docx [file pone.0345642.s001.docx]

Below is our point-by-point response to each of the reviewers' comments. Corresponding revisions in the manuscript are indicated in each response using line numbers or equation numbers. In the manuscript, all textual changes are highlighted in red.

**Reviewer 1**:

**Subjective suggestions :**

1. **The introduced model is highly complex and has a huge number of parameters, whereas the desired effects could have been displayed with much less effort.**

**For example.**

a. **considering the short time span, effects like natural deaths and births could have been neglected.**
b. **vaccinations could have been left out without drastically changing the general dynamics of the model.**

**This would have made the model significantly simpler and would have left space for a more rigorous analysis of outcomes other than “active cases”.**

Response: We agree that births and natural deaths are often excluded from short‑term epidemic models; however, adding them here introduces only a few extra parameters, while most complexity arises from our immunity‑ and decision‑based compartments. Thus, we keep them for structural consistency and to facilitate future extensions to longer horizons where demographics may become important.

Regarding the reviewer's comment on vaccination inclusion in our model, we agree that models should indeed remain as simple as possible. However, vaccination behavior is a primary driver of the dynamics we seek to explain, and omitting it would remove a core feedback loop. Our framework distinguishes partially immunized individuals from susceptible individuals, with immunity upgraded by primary vaccination, booster doses, or infection-induced recovery. Because vaccinated individuals can still become infected, their risk perception—and thus testing and revaccination choices—evolves: Individuals who received their primary series some time ago respond more actively to seek care based on current prevalence (rate F1), whereas booster recipients are driven more by perceived disease severity (rate F2). These dynamics create essential interactions between immunity, behavior, and transmission that would be lost if vaccination were excluded from the model.

2. **Less emphasis could also have been placed on model fitting to COVID-19 real numbers without reducing the significance of the paper – on the contrary, the field of application of COIVD-19 could have been extended to a potential future disease X which is currently much more important to the scientific community and the decision makers. However, since this paragraph is only my personal opinion, I leave it to the authors to take it into account or not.**

Response: We appreciate the reviewer’s thoughtful suggestion regarding the broader applicability of our model. Although we used COVID‑19 as a case study due to the availability of real-world data for model calibration and validation, the model’s core feature is its behavioral feedback tied to evolving immune status, from a susceptible population early in an outbreak to one with widespread vaccination. Capturing how behaviour shifts as immunity accumulates is directly applicable to future pandemics with any emerging pathogen, especially when vaccines or other countermeasures become available mid‑course. We appreciate this perspective and will consider emphasizing the model’s adaptability in future work.

**General concerns**

1. **Two concerns about the specification of the compartment model, to be precise, with the specification of the IT1, IT2 and H compartments**
a. **To me, one of the most interesting features of compartmental models/system dynamics models is, that rates can be interpreted as probabilities and delays (and any mixture of them) at the same time. For parametrization though, it is crucial to distinguish these two. Thus, it is good modelling standard in compartment modelling, that, whenever the flows out of a compartment split, a splitting probability is introduced -> precisely how it is done in E1-> A1/I1. However, this is not done for e.g. A1-> IT1/R. This results in the problem that rho (in your specified model) does not stand for a recovery-rate but rather for the recovery rate multiplied by the chance of not being tested as an asymptomatic - which makes it difficult to interpret. Note that you have the same problem for I1->IT1/R, for IT1 -> R/H and, of course, for the second infection cycle.**

Response: Thank you for highlighting this important modeling issue, particularly its implications for interpreting recovery rates. In response to your insightful comment, and as further elaborated in your point (b), we have revised the model structure to improve clarity and consistency in representing flows out of the infectious compartments.

Specifically, we introduced an additional compartments, IU1 (in primary dynamics) and IU2 (in secondary dynamics), to explicitly represent undetected asymptomatic and symptomatic individuals. With this modification, individuals in compartments A1 (asymptomatic) and I1 (symptomatic) now move to either IT1 (tested) or the new IU1 (untested) compartment, with the split governed by clear probabilities (see revised Fig. 1, p 6, and explained in lines 77 -80.). We assume that individuals in A1 remain in that compartment for a longer average duration (parameterized by a1) compared to I1, before testing occurs. However, both durations are assumed to be short enough that neither recovery nor death occurs within these compartments. Moreover, we assume that individuals in compartments A1 and I1 do not contribute to transmission, as these compartments represent a pre-testing stage with a short duration of stay, during which the likelihood of onward transmission is assumed to be negligible (lines 114 - 120). We have applied the same modeling modification to the second dynamics.

Regarding the transition from IT1 (IT2) to R and H, we have retained the original structure, as we consider recovery and hospitalization to be governed by distinct biological processes, each with its own independent rate. This approach, which avoids the use of a splitting probability in this specific case, is supported by several previous studies, for example:

- From identified infected compartment to Hospitalized and Quarantined compartment without splitting probability [1]
- From carriers(C) to ill (I) and recovered (R) compartment[2]
- From Infected (I) to Hospital (H) and Recovery (R) [3]

We believe these changes clarify parameter interpretation and align the model with standard compartmental conventions while preserving its biological realism.

b. **The property of being detected influences the recovery of an individual which is (to me) unreasonable.	Even if the average durations are chosen, so that the recovery time for the tested is, on the average, equivalent with the untested (something like 1/T1 + 1/delta_t = 1/delta), the underlying time distributions differ (the sum of two exponential distributions is not an exponential distribution). Concern (b) is not a big problem for the IT. -> R/H split, since different recovery times for hospitalized are reasonable (one should think of concern (a) though). However, for the others, something more rigorous needs to be done. The simplest way around would be to add an additional IU1 compartment meaning that all A1 and I1 individuals always flow into IT1 or IU1 first, before they are hospitalized or recover.**

Response: Thank you for raising this point. We agree that if detection status is allowed to alter recovery dynamics, inconsistencies can arise in the underlying dwell‑time distributions. Our revision—introducing the **IU1** compartment (see response a)—resolves this by routing every individual in **A1** or **I1** first to **IT1** (tested) or **IU1** (untested) before any recovery or hospitalisation step, with the same change applied in the secondary dynamics. This structure allows recovery and hospitalization processes to be governed by consistent and independent rates, regardless of detection status, and avoids the problematic assumption that detection directly alters the recovery time distribution. We greatly appreciate your insightful recommendation, which significantly improved the conceptual rigor of the model.

2. **As far as I understood, the choice of the message function is rather arbitrary and somewhat biased. By definition of the function, g1 is always by far larger than g2 meaning that increasing prevalence will always have a much larger impact than increasing hospitalized and deaths. Moreover, deaths enter the functions only via new deaths per day whereas all other quantities enter as active cases which is not fair either. In general, I would argue, that knowledge about (a) an infected (b) a tested infected (c) a hospitalized and (d) a death individual will not only have a different information coverage (e.g. newspapers cannot write about non-tested individuals) but also a different impact w.r. to adherence (increasing death tolls are likely much more influential than increasing case numbers). The authors themselves wrote about this problem and that the model produces counterintuitive results compared to other studies, yet they do not seek for a reason within in their model assumptions.**

Response: Thank you for highlighting the imbalance between our two information “message” functions g1 (prevalence) and g2 (severity). Because g1 formerly used both detected and undetected symptomatic cases, it was systematically larger than g2, which relied only on deaths and hospitalizations; this could overstate the behavioral weight of prevalence relative to severity.

To correct this, we made the following two changes in the revised manuscript

i. We have redefined g1 to depend solely on publicly visible indicators-- tested cases and hospitalizations (Equation 4)
ii. We assumed the reactivity to severity is twofold of the reactivity to prevalence- such assumption reflects the notion that severity-related events are generally more visible to the public and psychologically impactful, often prompting stronger behavioral responses ( lines 306 – 316)

These modification address somehow the dominance of g1 over g2. In general, the limitation that message functions formulation and fixing baseline values to reactivity parameter are based on only theoretical basis is acknowledged (lines 619 – 629)

A sensitivity check varying the severity‑reactivity parameter across a wider range shows the revised formulation reduces g1’s dominance without materially altering peak‑infection projections, thereby improving conceptual balance while preserving overall model behavior.

Regarding the discrepancy between our model and other studies, we acknowledge that varying definitions of the message functions in our model can shift the dominant behavioral drivers and lead to different outcomes as compared to other studies. To make this clear, we have added a brief discussion (lines 595 -599) to provide further context and transparency regarding the assumptions behind our model’s structure could be one the sources of the discrepancy.

**Specific comments:**

1. **25) Considering potential publication dates, the first sentence should be updated.**

Response: Thank you for the suggestion. We have updated the reference and replaced it with a more recent publication to reflect the most current literature and ensure the relevance of the citation (line 4).

2. **62) The author have properly highlighted several papers in which human adherence/compliance is incorporated. Please explain, how your approach differs from or relates to theirs, respectively.**

Response: Thank you for prompting a clearer comparison. Like earlier information‑index models, our framework links behaviour to epidemic indicators, but prior work typically treats the population as homogeneous and ties compliance to a single signal—most often prevalence. Our model extends this paradigm in two ways: (i) it distinguishes two information streams—prevalence (g1) and severity (g2)—and allows separate reactivity parameters, and (ii) it embeds heterogeneity by immune status, so vaccinated/boosted and non‑immune individuals can weigh those signals differently. These additions generate richer, more realistic behavioural dynamics while remaining within the information‑index tradition. The revised manuscript now outlines this distinction in lines 40‑56.

3. **Figure 1.) Where does the flow for the hospitalized originate in your sketch? It becomes clear after looking at the formulas, but should already be clear when looking at the picture.**

Response: Thank you for noting this. In the diagram (Figure 1), arrows from both tested compartments—IT1 in the primary cycle and IT2 in the secondary cycle—feed into the Hospitalised (H) compartment. We have re‑checked the figure and confirmed that these pathways are indeed present and correctly labelled.

4. **132) "<...> regardless of the information." Please specify "the information" in more detail here. I would even suggest to start the section by defining the term "information" and how it is modelled (cal. N and V). This would help understanding how the rates are specified/modelled.**

Response: Thank you for the suggestion. We have added a new subsection, “Information,” immediately before the vaccination and testing rate definitions (lines 142 - 170) to explicitly define the information signals that govern behavioural rates in the model.

5. **136) "These rates are represented by a constant rate." Sentence sounds strange/unnecessary**

Response: Thank you for pointing this out. We agree that the original sentence was unclear and potentially unnecessary. Our intention was to clarify that the mandatory components of vaccination and testing rates are modeled as constants, while the voluntary components are dynamics. In the revised manuscript, we now made it clear by only adding a phrase ‘modeled as constant’ as in the sentence ‘First, the mandatory vaccination and testing rates (modeled as constant) represent the rates for the portion of the population that will be vaccinated or tested regardless of the information.’ (line 173)

6. **163) As far as I understand the formula, the term "average delay <..> to reach the <..> public" is not semantically correct. It rather represents the memory of the general public w.r. to earlier observations - the smaller a, the longer the memory. While this, to some extent, also acts like a delay - the better the public remembers the past, the less important are more recent developments - it should not be confused with a delay: the most recent observations will always influence most (x=0 is the maximum of e^(-ax)). To include an actual delay, a convolution with a Gaussian kernel instead of the exponential would be better suited. [However, I guess, in this case transformation (9) would not be possible though]**

Response: Thank you for the helpful comment. We now describe parameter a as a memory‑decay coefficient: smaller values mean the public retains past information longer, while larger values indicate faster forgetting. This usage follows earlier information‑index studies [4]. The revised wording appears in the Information Indices section (line 150).

7. **178) "where the upper dot denotes the time derivative" seems unnecessary considering the target audience of the paper**

Response: Removed in the revised manuscript

8. **eq (10)) please add V and N as arguments to F1, T1, F2, T2 to make clear how the equations are intertwined.**

Response: Thank you for the suggestion. We have updated Eq(10) accordingly in the revised manuscript.

9. **Section 3) In general, some of the introduced parameters in the model specification feel unnecessary, in particular when contrasting them with Table 1. E.g. as far as I understood there is no need to distinguish between B,D,tilde B and tilde D.**

Response: Thank you for the comment. Although the model originally introduced four reactivity parameters (B, D, $\tilde{B,}$ and $\tilde{D}$ ), we lacked data to estimate them separately, so we treated them as identical. In the revision, we have collapsed them into just two parameters—reactivity to prevalence (D) and to severity (B)—as shown in Eqs. 6–9.

10. **242-243) Apparently, birth and death rates have different units. I assume, death rate is per inhabitant, birth rate is in persons per day?! Please state.**

Response: Thank you for pointing this out. We have updated the units for clarity in the revised manuscript. Specifically, the birth rate is now stated as "individuals per day," and the death rate is specified as "per person per day" (see line 282-283).

11. **245) What does the number 1382042 represent? Does it refer to the maximum theoretical number of available doses per day, the highest ever recorded vaccinations per day or something else? Similar for the tests: considering test-kit availability, is really realistic that 0.5*55M ~ 22M tests are evaluated per day in South Korea?**

Response: We apologize for the confusion. We have clarified two points in the revised manuscript. First, the figure 1 382 042 now explicitly appears as South Korea’s daily maximum record for administered COVID‑19 vaccine doses obtained from the data in Our world in [5] (line 286). Second, our testing rate applies only to infectious individuals. There the maximum testing rate (Tmax) value (e.g 0.5) denotes a theoretical testing peak that can be achieved when public risk perception is highest. After updating the model based on reviewer’s comments and recalibration, the parameter has been adjusted to Tmax = 0.8, in the revised manuscript (lines 300‑305). Thus, under high information circulation, up to 80 % of infectious individuals could be tested per day, although the simulated peak rarely exceeds 0.4 in the baseline (doesn’t exceed 0.6 in scenarios by varying prioritization, Figure 7). In the model’s base case, them maximum number of daily cases reaches 1.4 million. Thus, a value of Tmax = 0.4 implies a daily testing rate of 40% of 1.4 million or 560,00 tests per day. This is consistent with observed data, where the maximum number of detected cases per day reaches around 600, 000, as shown in Figure 4.

12. **Table 1) I find the recovery rate of 1/14 and latency rate of 1/3 hard to believe for Spring 2022 and the prevalent Omicron variant.**

**Table 1) Please add units!**

Response: We set the recovery rate to 1/14 day⁻¹, reflecting the widely used 14‑day mean infectious period for COVID‑19, a value still employed in Omicron‑era models[6] . For the latent period we used 1/3 day⁻¹, consistent with sources cited in the manuscript and with South‑Korean estimates of a 3.5‑day mean incubation for Omicron [7].

Units have been added to Table 1 as suggested.

13. **312) "The result in Figure 3 shows that the model best estimates the observed daily cases when voluntary vaccination and testing are considered." How is this surprising? As far as I understood, the model parameters were fitted to the "responsive" parameters?!**

Response: Thank you for the observation. The reviewer is correct that the model was calibrated solely with the behavioral (responsive) parameters that account for the effect of voluntary vaccination and testing. Our earlier wording—meant to contrast behavioral (including responsive parameters) and non‑behavioral (excluding responsive parameters) scenarios—could be confusing as implying that both versions were calibrated and compared on daily cases. To avoid that confusion, we have removed the non‑behavioral curve from Figure 3. In addition to demonstrating the model’s calibration to cumulative cases (Figure 2, panel a ), Figure 3 demonstrates that it also fits to the daily case trajectory. We updated the corresponding description accordingly (lines 366–372).

14. **318-322) I don't quite understand, how the effective reproduction number in the fitted model can be smaller than one. If it was, in theory, there should not be a disease wave?! Note: the computed Re should not only depend from the parameters (as stated) but, above all, the initial conditions. Moreover, comparing the computed Re from the model, which is a momentary measure at the start of the simulation, with the "mean reproduction number" from observational studies does not make sense to me.**

Response: After model revisions and recalibration, the effective reproduction number is Re = 1.24 (line 373-374). Equation 13—derived at the disease‑free equilibrium—depends solely on model parameters, not on the transient initial conditions of the simulation; Variables such as S10, S20, V10, and V20 denote the disease-free equilibrium values of the respective compartments, which are determined based on the model parameters (as defined in Equation 12 (clarified at line 243-244). We have also updated Figure 2: the stability map now uses the mandatory‑testing parameters T10 and T20 rather than the vaccination parameters, because testing, not mandatory vaccination, has the more substantial influence on Re in the current calibration. With the baseline parameter set, the disease‑free state is unstable (Re > 1), and, over an extended horizon, the system converges via damped oscillations to an endemic equilibrium; the initial rise and decline in incidence during the study period are driven by vaccination uptake and intensified testing.

15. **368) From my intuition is not clear to me, why reactivity to information about prevalence should have a much larger effect than the one for severity. I guess, this originates from the message function issue stated earlier.**

Response: We agree that the model’s heightened sensitivity to prevalence‑reactivity reflects the design of the message functions. Because prevalence indicators (influenced by a number of tested and hospitalised cases) generate a far stronger signal than severity indicators (influenced by a number of hospitalized and deaths), adjustments to their reactivity coefficient have a larger behavioural impact. This clarification has been added at lines 396–402 of the revised manuscript.

16. **Figure 5/6) I understand, that the "active cases", as defined by I1+I2+A1+A2, is important to analyze disease containment, since those are the ones most influential for the spread. However, the transmissibility of the asymptomatic is smaller than the one for the infected whereas (1-delta) of the detected infected individuals contribute to spreading as well (and with a higher test adherence, this part would increase). So the chosen outcome is in my opinion not optimal to evaluate the "success" of an intervention. Why not take the peak of the hospitalized compartment or the cumulative deaths instead? From the public health aspect this would be more influential anyway.**

Response: We appreciate the reviewer’s thoughtful observation. Because our goal is to understand how information‑driven voluntary behaviour shapes transmission, we track the peak of ‘active cases’ (now undetected cases- IU1 and IU2) as a proxy for infectious pressure; individuals in (1 – δ)(IT1 + IT2) and H are assumed non‑transmitting. Peak hospitalisations and cumulative deaths are valuable public‑health metrics, but in our framework greater voluntary testing naturally produces more detected cases and, in turn, more hospitalisations. Thus, our model aims to elucidate how information‑responsive behaviour shapes epidemic trajectories rather than to quantify intervention effectiveness by reductions in hospitalisations or deaths. Nonetheless, our model with the behavioural‑feedback framework preserves the findings of conventional model that greater care‑seeking to vaccination and testing reduces overall prevalence.

17. **478) "even the" -> "even IF the"**

Response: Corrected

18. **578) Additional delay time of vaccinations i.e. time between the shot is issued and the vaccination works properly (~14days for typical CoV MRNA vaccines) should be added to the limitation list.**

Response: Thank you for the suggestion. The revised manuscript now explicitly acknowledges that our model omits the real‑world delay between vaccination and the development of immunity. (lines 642–646)

**Reviewer 2:**

**This manuscript presents a timely and technically interesting behavioral transmission model of COVID-19, calibrated to data from South Korea during the Omicron wave (February–May 2022). The study makes a valuable contribution by incorporating immunity-based behavioral responses into a compartmental modeling framework and assessing their impact on epidemic dynamics. The use of real-world data for model calibration and the exploration of behavioral heterogeneity adds to its relevance for both public health policy and epidemic modeling literature.**

Response: We sincerely thank the reviewer for the thoughtful and encouraging comments. We are pleased that the reviewer finds our study timely, technically sound, and a valuable contribution to the modeling of behavioral responses in the context of COVID-19. We especially appreciate the recognition of our efforts to incorporate immunity-based behavioral heterogeneity and the use of real-world data in model calibration. These aspects were central to our motivation, and we are glad they have been acknowledged.

References

[1]	S. R. Bandekar and M. Ghosh, “Mathematical modeling of COVID-19 in India and Nepal with optimal control and sensitivity analysis,” *Eur. Phys. J. Plus*, vol. 136, no. 10, p. 1058, Oct. 2021, doi: 10.1140/epjp/s13360-021-02046-y.

[2]	B. Buonomo and R. Della Marca, “A behavioural vaccination model with application to meningitis spread in Nigeria,” *Appl. Math. Model.*, vol. 125, pp. 334–350, Jan. 2024, doi: 10.1016/j.apm.2023.09.031.

[3]	O. Koutou, A. B. Diabaté, and B. Sangaré, “Mathematical analysis of the impact of the media coverage in mitigating the outbreak of COVID-19,” *Math. Comput. Simul.*, vol. 205, pp. 600–618, Mar. 2023, doi: 10.1016/j.matcom.2022.10.017.

[4]	B. Buonomo and R. Della Marca, “Effects of information-induced behavioural changes during the COVID-19 lockdowns: the case of Italy,” *R. Soc. Open Sci.*, vol. 7, no. 10, p. 201635, Oct. 2020, doi: 10.1098/rsos.201635.

[5]	E. Mathieu *et al.*, “Coronavirus Pandemic (COVID-19),” *Our World Data*, Mar. 2020, Accessed: Apr. 17, 2025. [Online]. Available: https://ourworldindata.org/coronavirus/country/south-korea

[6]	Y. R. Kim, Y. Min, and J. N. Okogun-Odompley, “A mathematical model of COVID-19 with multiple variants of the virus under optimal control in Ghana,” *PLOS ONE*, vol. 19, no. 7, p. e0303791, Jul. 2024, doi: 10.1371/journal.pone.0303791.

[7]	Y. Liu, S. Zhao, S. Ryu, J. Ran, J. Fan, and D. He, “Estimating the incubation period of SARS-CoV-2 Omicron BA.1 variant in comparison with that during the Delta variant dominance in South Korea,” *One Health*, vol. 15, p. 100425, Dec. 2022, doi: 10.1016/j.onehlt.2022.100425.
