# Supplementary material for: The impact of human behavioral adaptation stratified by immune status on COVID-19 spread with application to South Korea
Source: PLoS One. 2026 Apr 6;21(4):e0345642. doi: 10.1371/journal.pone.0345642 (PMC13052854; doi:10.1371/journal.pone.0345642)
Supplement: Attachment — Submitted filename: Reply letter to reviewers comments_FINAL.docx [file pone.0345642.s003.docx]

*The manuscript has been revised to address the reviewers’ major comments by strengthening methodological clarity, transparency, and presentation. Key revisions include: (i) clearer discussion of model scope, assumptions, and limitations, particularly regarding behavioral responses, applicability across epidemic versus endemic phases, and the exclusion of multiple co-circulating variants; (ii) improved parameter estimation and uncertainty quantification through the addition of 95% confidence intervals for fitted parameters and a dedicated sensitivity analysis subsection describing the methods used; (iii) inclusion of a new Scenario subsection in the Methods to clearly define and organize the simulated behavioral and policy scenarios; and (iv) enhanced model validation and presentation, including quantitative goodness-of-fit metrics, clarification of daily testing dynamics, correction and reformatting of figures and tables, and the addition of a complete table of symbols and parameters. References were updated and the manuscript was thoroughly proofread to improve clarity and consistency.*

*Below, we provide a point-by-point response to each reviewer comment. All new changes introduced in the present revision are highlighted in* ***blue****, while modifications made during the first revision are retained and shown in* ***red*** *in the manuscript.*

Reviewer-3

The presented work is interesting and unifies several ideas of extensions of SIR type ODE models to a 15 compartment SEIR type model that partitions the population into a (naive) primary and a (partially immune) secondary branch. Vaccination and testing rates each comprise a constant mandatory component and a voluntary component governed by two information indices for prevalence and severity. The parameters are estimated by data of the Republic of Korea from 1 Feb to 31 May 2022 and explore how information related parameters influence epidemic peaks. In contrast to agent-based models of the whole population this approach allows more analytic analyses which are presented nicely. However, there is also the drawback of less flexibility (preventing state explosion) and the formulas lack the co-existence of several strains, which for example was especially relevant when the Omicron BA.1 and BA.2 strain emerged (which falls into the period of the collected data), leading do very different immune response behaviour (both after infection and vaccination) for the affected population groups. The quality of the paper could be increased if these and other limitations, and what they mean for policy makers, would be discussed in more detail.

**Reply:** *We sincerely thank the reviewer for the insightful and well-considered feedback. We agree that incorporating co-circulating variants (such as Omicron BA.1 and BA.2) and additional immunity states can enhance realism, but doing so also substantially increases the number of compartments and parameters (e.g., strain-specific infection, recovery, and immune evasion rates). Given the primary focus of this study was to isolate the impact of behavioral adaptation based on immune status (i.e. How information-driven voluntary vaccination and testing behaviors shape epidemic dynamics through differential responsiveness among immunologically naïve and partially immune individuals), we opted for a parsimonious structure to maintain mathematical tractability and avoid the uncertainty associated with parameterizing multiple co-circulating strains without granular strain-specific behavioral data.*

1. 262–269: Please provide seroprevalence or administrative vaccination coverage data to substantiate the 85 % figure on 1 Feb 2022. Sensitivity analysis in Table 1 varies biological parameters but not this initial condition, but Figure 3 shows the model dynamics are highly sensitive to the susceptible fraction. This uncertainty is even more prevalent as the calibration period falls directly in the early (and in some countries double-) Omicron-waves.

**Reply:** *First of all we would like to clarify that Table 1 reports baseline parameter values and does not represent a sensitivity analysis. Figure 3 illustrates the model fit to observed daily cases rather than parameter sensitivity. We have added a reference supporting the assumption of an 85 % susceptible fraction on 1 Feb 2022, based on seroprevalence study in South Korea (Line 313-324). However, we acknowledge the uncertainty of vaccination coverage of 85% (fraction of initial partially immune population (S2+S3) ranging from 70% to 98%) with the waning immunity), and added this into sensitivity analysis (Fig 8) and corresponding explanation in the method section (lines 427-429).*

2. Only four parameters are fitted using vaccination and testing data. Because mandatory and voluntary rates are both fitted indirectly, their effects are confounded. Please provide confidence intervals or further distributions to validate the fitted parameters.

**Reply**: *Thank you for this constructive comment.* Although mandatory and voluntary rates jointly shape vaccination and testing dynamics, they enter the model as additive processes that cannot be independently identified from aggregate data. Our fitting process therefore focuses on estimating the combined effect captured by the four fitted parameters (e.g. $\beta_{1}, \beta_{2}, k, \xi$). *In the revised manuscript, we now report 95% confidence intervals for all fitted parameters (See Table 2). These intervals were calculated using the covariance matrix produced by the nonlinear least-squares fitting algorithm (curve_fit), which provides an estimate of the uncertainty surrounding each parameter. A brief explanation of the procedure has been included in the Model fitting and parameter estimation section (Lines 365–368).*

3. In equation (13) voluntary testing does not appear, even though the force of infection depends on the isolated fraction, therefore the following results may not be true, so either include this fraction or explain why the simplification is legitimate.

**Reply**: *We appreciate the reviewer’s comment. Because the next-generation matrix is computed at the disease-free equilibrium, the information variables of voluntary vaccination/testing terms evaluate to zero (* $V=N=0$*), in prevalence-dependent functions and the testing/vaccination rates reduce to their baseline constants (T10, T20 or F10, F20) in estimating the effective reproduction number* $R_{e}$*. We have added a detailed explanation to the revised manuscript (Lines 227–236).*

4. Figure 2b shows the fitted model results, however it is hard to assess the goodness of fit without metrics, so provide RMSE or another quantitative fit. Also, include observed vs. fitted daily testing numbers.

**Reply:** *We thank the reviewer for this constructive suggestion. We would like to clarify that the reviewer is referring to the version of the manuscript submitted during the first review cycle, in which the fitting results appeared as Figure 2. In the revised manuscript (submitted after the first round of review), substantial restructuring was done, and the corresponding results now appear in Figure 3. In the revised manuscript, we now explicitly quantify the goodness of fit by reporting normalized RMSE values (based on min–max scaling) for each data stream. The resulting normalized RMSE values (0.022 for cumulative cases, 0.091 for vaccinations, and 0.015 for deaths.) indicate that the model attains comparable relative accuracy for cumulative cases and deaths, while the fit to cumulative vaccination data is somewhat less precise (Lines 368–375). Nonetheless, the overall fitting performance remains strong, and the model successfully reproduces the major temporal patterns across all three data sets despite differences in scale.*

*Regarding daily testing, we note that the cumulative case fitting is inherently based on daily detected cases (*$I_{T1}+I_{T2}$*). As presented in the manuscript, Figure 4 shows the observed daily tested cases along with the corresponding model approximation. This figure illustrates the temporal pattern of model-predicted daily testing and demonstrates how the model reproduces fluctuations in detected cases over time, complementing the cumulative fitting shown in Figure 3a.* In the revised manuscript, we have clarified this point to clearly indicate how Figure 4 complements the cumulative case fitting presented in Figure 3a (Lines 390-397).

5. Some statements need more explanation or clarification when they are true, for example “increasing reactivity from 50 % to 100 % would reduce the peak by 16 %” is only true under the assumption of fixed mandatory rates. Also, reactivity is not an intervention, but a behavioural parameter, thus confidence intervals (or other measures of uncertainty) are recommended.

**Reply:** *We thank the reviewer for this helpful comment. It appears that the reviewer is referring to an earlier version of the manuscript, in which the illustrative example reported a reduction of 16% when increasing the reactivity of partially immune individuals from 50% to 100%. In the revised manuscript, this result has been updated based on the corrected simulations and now states that such an increase leads to a 15% reduction relative to the baseline, and a 33% reduction compared to the scenario in which partially immune individuals do not react to information at all (θ = 0). We agree that the effects of the voluntary components are observed under the assumption of fixed mandatory rates. To clarify this, we have revised the discussion section to explicitly state the conditions under which these effects occur. Additionally, we note that uncertainty in this behavioral parameter is explored through parameter variation and further assessed via one-way sensitivity analysis (Lines 583–593).*

6. There are either some typos, the paper should be proof-read once more.

**Reply**: *We thank the reviewer for this observation. The manuscript has been carefully proofread and revised to correct typos and improve overall clarity.*

7. Provide a complete table of symbols.

**Reply**: *We appreciate the reviewer’s comment. In the revised manuscript, we have now added a complete and consolidated table listing all compartment symbols and parameters along with their descriptions (Table 1) and we provided the parameter’s baseline values in a separate Table (Table 2). Table 2 also reports parameter ranges, including 95% confidence intervals obtained from the literature when available and from the model-fitting procedure, as well as ranges defined as ±25% of the baseline values used in the sensitivity analysis. We believe this addition improves clarity and makes the model structure easier to follow.*

8. The assumption from [34] that the number of exposed people is 20x the number of infected people seems wrong. The source [34] is not the initial source, [34] references to sources from early 2020. Other simulation studies use much lower ratios for exposed to infected. Please verify that or explain that assumption.

**Reply**: *We thank the reviewer for pointing this out. We acknowledge that this ratio could be higher than in some other modeling studies, which often use lower exposed-to-infected ratios or treat the initial exposed population as a free parameter.* In our case, we intentionally selected a larger initial exposed population to account for the possibility of substantial undetected or presymptomatic infections at the start of the simulation. Although this choice leads to an overestimation of the initial exposed compartment, the model is initialized with zero undetected infectious individuals; therefore, the large exposed count naturally transitions into undetected infections during the early phase of the simulation. We have clarified this rationale in the revised manuscript (Lines 329–337).

The manuscript is promising but requires some methodological clarification and stronger justification of assumptions. Also, it needs clarification under which prerequisites and in which phase of the pandemic their results are applicable for policy makers, including a discussion of the limitations, especially after entering the endemic phase.

**Reply:** *We thank the reviewer for this insightful comment. In the revised manuscript, we have clarified the specific context and prerequisites under which our results are applicable namely, epidemic-wave periods characterized by rapid transmission, active testing, and dynamic behavioral responses. We have also added a brief explanation regarding the more limited quantitative applicability of our findings during the stable endemic phase. These points are now explicitly noted in both the Discussion and the Limitations sections to better guide interpretation of our results (Lines 557-563, 605-611, 702-706).*

Reviewer 4

This study develops a behavioral transmission model to examine how COVID-19 spread differs between partially immune and susceptible individuals. Using data from South Korea (February–May 2022), the model incorporates variations in vaccination response, testing behavior, and reliance on information about disease prevalence and severity. Simulations show that if partially immune individuals react to information as strongly as susceptible individuals, peak active cases may
decrease by 16%. However, shifting their risk perception from prevalence to severity could increase the peak by 50%. These results emphasize the importance of adaptive vaccination and testing strategies as public risk perceptions change in the post-pandemic era. The paper has the following issues:

**Reply:** We would like to clarify that *it appears that the reviewer is referring to an earlier version of the manuscript, in which we reported a reduction of 16% when increasing the reactivity of partially immune individuals from 50% to 100%. In the revised manuscript (submitted during the first-round revision), this result has been updated based on the corrected simulations and now states that such an increase leads to a 15% reduction relative to the baseline, and a 33% reduction compared to the scenario in which partially immune individuals do not react to information at all (θ = 0). Moreover, shift in the risk perception from prevalence to severity increases the peak by 57%.*

1. Table 1 contains several errors

**Reply:** While the specific errors referred to were not clearly identified, we carefully revised Table 1 and the associated parameter list. In doing so, *we identified a few inconsistencies between the parameter (*$F_{10}, F_{20}, T_{10}, T_{20}$*) values reported in the text and those presented in the table. These have now been corrected. In the revised manuscript, we also improved the table formatting for clarity and reorganized the parameter and symbol descriptions into a separate table (Table 1), following a suggestion from another reviewer and parameter values are reported in a separate Table (Table 2). We hope these revisions resolve any confusion the reviewer may have encountered.*

2. Figure 4 appears to be incorrect.

**Reply**: *We thank the reviewer for this comment. However, based on the general comment of the reviewer it appears that the reviewer is referring the original submission prior to the first revision. Therefore, Figure 4 presented the stability region analysis, which was renumbered as Figure 2 during the first revision. Although the specific issue with Figure 4 was not explicitly identified, we carefully re-examined the figure (Figure2 panel a-modified stability figure-during the first revision) and identified a resolution-related issue in the contour plot. Specifically, the initial contour resolution of 20 levels did not fully capture the maximum value of* $R_{e}$*at the baseline* $(T_{10},T_{20})=(0.2,0.2)$*reported in the text (*$R_{e}=1.24$*), resulting in a lower apparent maximum. This has now been corrected by increasing the contour resolution to 100 levels, ensuring that the value of* $R_{e}$*is accurately represented across the parameter space. Additionally, we validated the corrected stability region by showing that trajectories of disease prevalence behave as expected: declining to zero when* $R_{e}<1$*and converging to a non-zero endemic equilibrium when* $R_{e}>1$ *(In the revised version-Figure 2, panel b). This confirms the accuracy of the updated stability region in Figure 2.*

3. Figure 6 is not accurate and requires correction.

**Reply**: *We thank the reviewer for the comment. Although it was not clear to us which specific aspect of Figure 6 was considered inaccurate, we carefully re-examined the figure and its caption. We identified an error in the caption: the value describing the “level of reactivity by susceptible (non-immune) individuals” was incorrectly stated as 10. The correct values are* $D=50$ *for prevalence and* $B=100$ *for severity. This has been corrected in the revised version. We appreciate the reviewer’s comment, which prompted us to identify and fix this issue. We appreciate the reviewer’s comment, which helped us identify and correct this issue, and we would be happy to address any additional concerns should the reviewer provide further clarification.*

4. The overall English quality of the manuscript is very weak and needs thorough revision.

**Reply:** *We appreciate the reviewer’s observation regarding the quality of the English language in the manuscript. In response, we have thoroughly revised the manuscript to improve clarity and readability. The updated version has been carefully edited for language quality throughout. We hope the revised manuscript meets the reviewer’s expectations.*

5. The references are outdated; please update them with recent works from 2023–2025.

**Reply**: *We thank the reviewer for this comment. We have updated the majority of the references, particularly those published before 2022, by incorporating more recent studies from 2023–2025. We hope the updated reference list meets the reviewer’s expectations.*

6. The following references should be consulted and incorporated into the study:
   • DOI: 10.3934/math.2025986 ; DOI: 10.3390/mca30050100;
   DOI: 10.1371/journal.pone.0331243; DOI: 10.3390/math13172822

**Reply:** *Thank you for suggesting these additional references. The recommended papers focus on advanced analytical techniques for partial differential equations, including Lie symmetry methods, bifurcation analysis, and stability characterization. Our study primarily relies on numerical simulations because the high dimensionality and nonlinear structure of our model make a full analytical treatment challenging. We have now added a statement in the* Limitations *section noting that we did not investigate the analytical properties of the model and that methods such as those used in the suggested papers could be applied in future work to study bifurcation behavior around the threshold* $R_{e}=1$ *and other qualitative dynamical features. The references have been cited in this context (Lines 695-706).*

7. What is the role of θ in Figure 6? Please clarify its meaning and contribution to the analysis.

**Reply**: *We thank the reviewer for raising this point. In our model,* $\theta$*represents the reduced reactivity of partially immune individuals to disease-related information relative to susceptible individuals. We have clarified this in the revised manuscript. Specifically, we expanded the description in the results section to explain how variations in* $\theta$*influence behavioral responses and, consequently, the dynamics of active cases (Lines 476-479). We hope this clarification adequately addresses the reviewer’s concern.*

8. Explain the significance of the parameters F10, F20, T10, and T20 used in the figures. What do they represent, and how do they influence the model?

**Reply**: *Thank you for your comment. These parameters are already defined in the manuscript as the* mandatory *components of vaccination and testing rates (Page 9-10, lines 150-161). Specifically,* $F_{10}$*and* $F_{20}$*represent mandatory primary and booster vaccination rates, while* $T_{10}$*and* $T_{20}$*represent mandatory testing rates in the primary and secondary dynamics. These rates correspond to the portion of the population that undergoes vaccination or testing independent of information. To improve clarity, we have now added a brief statement explaining how these parameters influence the baseline level of uptake and help distinguish mandatory from information-driven voluntary behaviors (Line 162-164).*

Reviewer 6:

The paper describes a thorough epidemiological analysis of Covid-19 spread in South Korea, using multi-compartment models that incorporate the kinetics of transitioning from one compartment to another. Although I agree with reviewer 1 that the model is quite complex, I think the methods are quite detailed and could be informative. It looks like the comments from the previous review cycle were addressed adequately. The only other comment I had was for Table 1. All the parameter values indicated do not have any standard error associated with them. It could be worth addressing how deviations in the parameter value assumptions for those parameters, to which the model is especially sensitive to, would affect the results and/or its interpretation.

**Reply:** *We thank the reviewer for this thoughtful comment. In the original submission, we had included a one-way sensitivity analysis to quantify how variations in key parameters influence the cumulative incidence (Figure 8). In the revised manuscript, we additionally report* ***95% confidence intervals for all fitted parameters (Table 2)****, providing a measure of uncertainty associated with their estimated values. Together, the sensitivity analysis and the confidence intervals address how deviations in key parameter assumptions may affect the model outcomes and their interpretation.*
